# A two-stage spectral model for sound texture perception: Synthesis and psychophysics

**DOI:** 10.1177/20416695231157349

**Published:** 2023-02-22

**Authors:** Hironori Maruyama, Kosuke Okada, Isamu Motoyoshi

**Affiliations:** Department of Life Sciences, 13143 The University of Tokyo, Japan

**Keywords:** visuo-auditory interactions, texture, models, listening

## Abstract

The natural environment is filled with a variety of auditory events such as wind blowing, water flowing, and fire crackling. It has been suggested that the perception of such textural sounds is based on the statistics of the natural auditory events. Inspired by a recent spectral model for visual texture perception, we propose a model that can describe the perceived sound texture only with the linear spectrum and the energy spectrum. We tested the validity of the model by using synthetic noise sounds that preserve the two-stage amplitude spectra of the original sound. Psychophysical experiment showed that our synthetic noises were perceived as like the original sounds for 120 real-world auditory events. The performance was comparable with the synthetic sounds produced by McDermott-Simoncelli's model which considers various classes of auditory statistics. The results support the notion that the perception of natural sound textures is predictable by the two-stage spectral signals.

## Introduction

In the natural environment, we hear a variety of auditory events such as wind blowing, water flowing, and fire crackling. These textural sounds not only play an important role in auditory scene analysis and the perception of physical events (e.g., Gygi et al., 2004) but also provide a fundamental basis for the subjective richness of our auditory world. In contrast to the perception of speech and music, where not only phoneme features but also temporal order is decisive, the perception of sound textures is thought to be based on the statistical acoustic features over a certain temporal period (Attias & Schreiner, 1997; McDermott & Simoncelli, 2011; McDermott et al., 2013; McWalter & McDermott, 2018).

McDermott and colleagues have proposed a computational model of sound texture perception which is based on several classes of sound statistics (McDermott & Simoncelli, 2011; McDermott et al., 2013). Specifically, the model argues that marginal moments and pairwise correlations computed from subband envelopes decomposed from the original sound can characterize a particular sound texture. Importantly, the model is supported by strong evidence that a variety of natural sound textures can be synthesized from white noise by matching these statistics to the original sounds.

In fact, McDermott–Simoncelli's (MS’s) model of sound texture perception is inherited from Portilla–Simoncelli's (PS’s) model of visual texture perception (Portilla & Simoncelli, 2000). The PS model assumes that the perception of a visual texture is based on classes of low- and high-level image statistics, including moments in the orientation and spatial frequency subbands as well as autocorrelation/cross-band correlation within and across subbands. As with the MS model, the PS model can also synthesize a wide range of natural texture images by matching the PS statistics in a white noise.

**Figure 1.**
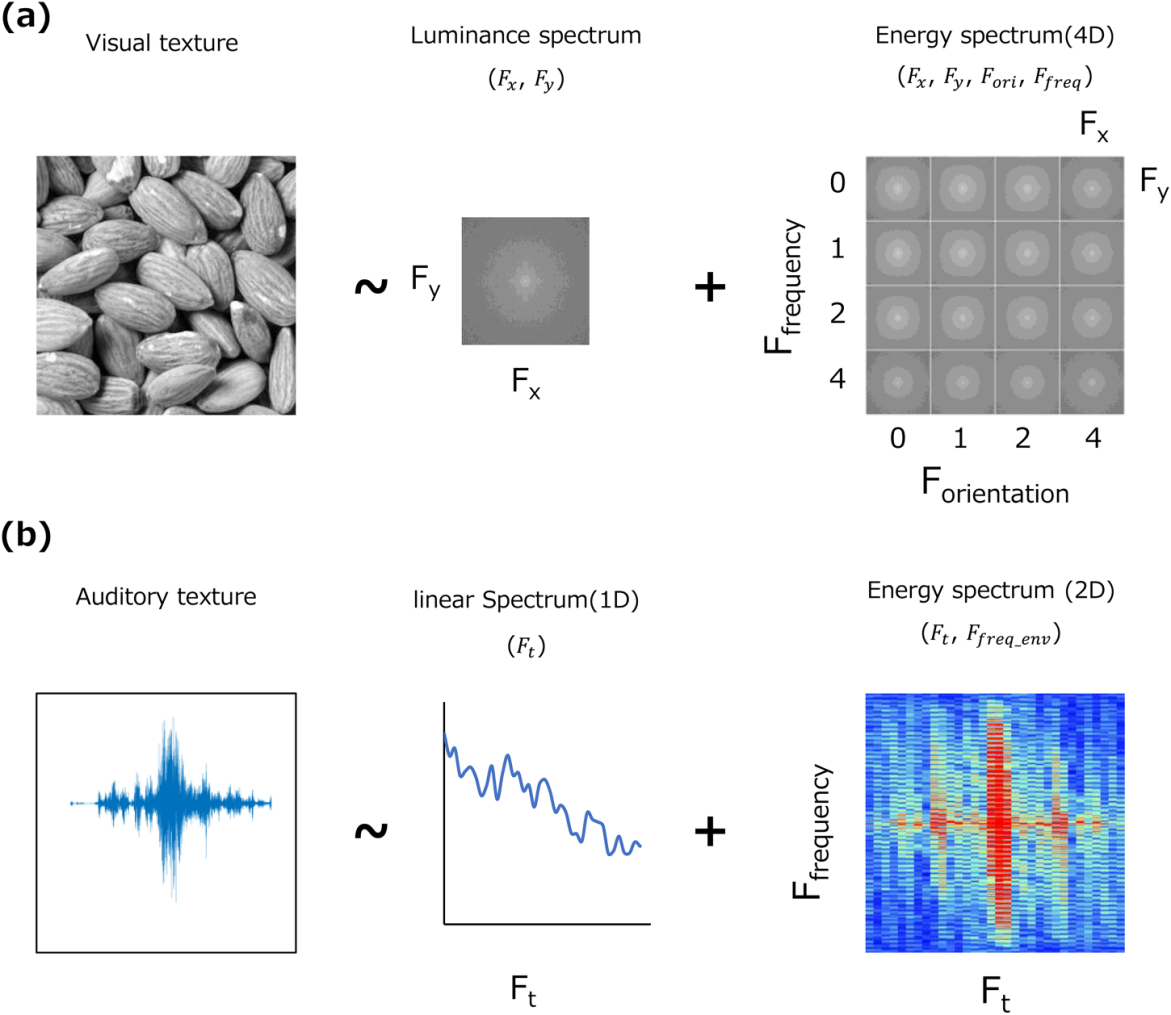
Two-stage spectral representation of visual and auditory textures. (a) Two-stage spectral representation of visual texture (2D spectrum of luminance and 4D spectrum of subband energy). (b) Two-stage spectral representation of auditory texture (1D spectrum of sound waves and 2D spectrum of cochlear subband envelopes). See text for details.

*Note*. 1D = one-dimensional; 2D = two-dimensional; 4D = four-dimensional.

As described above, both the MS model for sound texture and the PS model for visual texture have a complicated structure consisting of many classes of statistics. Recently, for visual texture, Okada and Motoyoshi (2021) showed that most of the PS statistics can be simplified by using a two-stage spectral representation (Figure 1a). The Okada–Motoyoshi model assumes that a visual texture is described by only two amplitude spectra (and pixel moment statistics): the two-dimensional spectrum of the luminance image (Fx, Fy) and the four-dimensional spectrum of the subband energy image (Fx, Fy, Fori, Ffreq). This spectral model, especially in its second stage, is considered a dimensional extension (from two-dimensional [2D] to four-dimensional [4D]) of the psychophysical Filter-Rectify-Filter model of visual texture discrimination (Bergen & Adelson, 1988; Bergen & Landy, 1991), as well as a Fourier-transformed representation of the auto/cross-correlation of subband energy in the PS model. Okada & Motoyoshi (2021) demonstrated the validity of their model by showing that a noise image that preserves the original two-stage spectra of natural texture (e.g., gravel) appears very similar to the original natural textures to a degree comparable to the PS-synthesized textures.

Since the perception of visual texture is explained by a simple two-stage spectral space, the perception of sound texture could also be explained by a simple two-stage spectrum by considering the MS statistics in the frequency domain. Specifically, part of the MS statistics can be represented by two amplitude spectra: the one-dimensional spectrum of the input sound—temporal frequency (Ft)—and the two-dimensional spectrum of its subband envelope—temporal frequency (Ft) × frequency of modulation frequency (Ffreq), as shown in Figure 1b. Similar two-stage spectral representations have been employed in the previous analyses of human and animal voices (Singh & Theunissen, 2003).

Can this two-stage auditory spectrum model (Figure 1b) account for the perception of various natural sound textures, as the two-stage spectral model of visual texture perception (Figure 1a) does? One of the strongest and ecologically valid demonstrations of a valid perception model is to synthesize a “metamer” stimulus that mimics the perception of the original natural stimulus (Freeman & Simoncelli, 2011; McDermott & Simoncelli, 2011; Portilla & Simoncelli, 2000). Here, we examine whether the two-stage spectrum model can synthesize a wide range of natural sound textures. The underlying idea is simple: noise sounds that match their two-spectrum original may be perceived as similar. If such is the case, then that would constitute strong evidence in favor of a two-stage spectral representation for the perception of natural sound textures.

### Sound Texture Synthesis Based on Two Spectra

**Figure 2.**
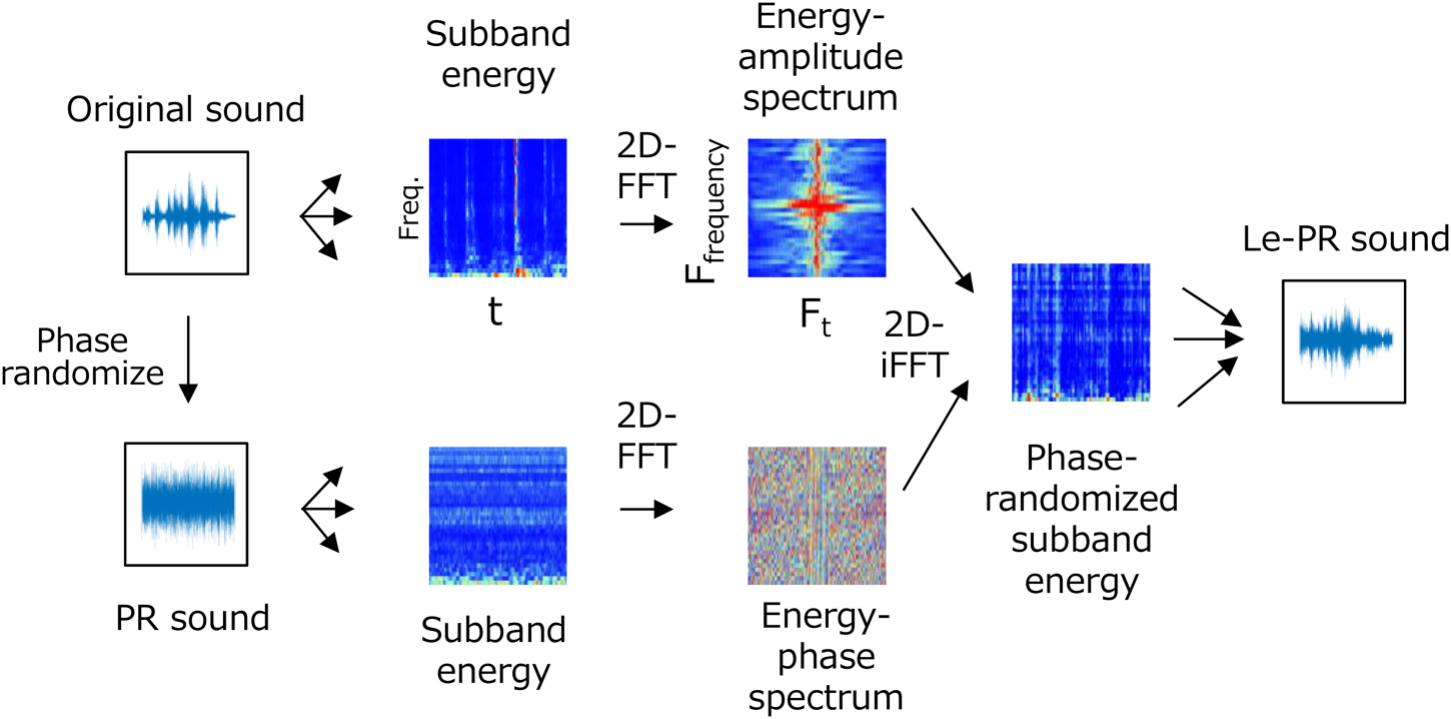
Schematic diagram of synthesized sound with two-stage spectral representation. PR denotes phase-randomized sound. le-PR denotes a phase-randomized sound that preserves the two-stage spectra. The original sound, PR, and le-PR are represented by waveforms. See text for details.

We synthesized sound textures using a method previously used in synthesizing visual images (Okada & Motoyoshi, 2021), namely randomizing phase while preserving two-stage spectra. The synthesized sound is generated by a simple procedure shown in Figure 2 (see Supplemental Material for demos). (1) A phase-randomized (PR) sound that preserves only the linear spectrum is obtained from the original sound. In the PR sound, only the linear spectrum of the original sound is preserved whereas the phase spectrum information is discarded and the white noise phase spectrum is used instead. As a result, the linear spectrum is preserved in the PR sound (as in the original sound), but the energy-amplitude spectrum is not. In the spectral calculation, the first and last 600 ms intervals of the signal (typically 3 s segments) were cosine tapered to avoid boundary artifacts. (2) Both the original sound and the PR sound are subjected to 30 band-pass filters, a high-pass filter, and a low-pass filter to obtain 32 filtered sounds and their energies. For computational efficiency, the energy is down sampled to 400 Hz. A compressive nonlinearity of 0.3 is then applied to the energy computations to simulate the cochlear transduction of sound. The bandpass filters are equally spaced in frequencies according to the equivalent rectangular bandwidth (ERB) N scale (Glasberg & Moore, 1990) that ranges from 20 to 10,000 Hz. Filter bandwidth was 3 dB or the equivalent bandwidth of the human ear. (3) The subband energy (envelope) of the original sound is obtained via the amplitude spectrum of the two-dimensional Fourier transformed. The phase spectrum, meanwhile, is obtained by a two-dimensional Fourier transform of the PR sound. The number of data points in the energy amplitude spectrum obtained by this 2D-FFT is 400 × 1/2 × s × 32, and the width of the bins is 400/(3 s × 1/2). (4) The resulting two-dimensional amplitude and phase spectra are then inverse-Fourier transformed to recover the random-phase subband energies while preserving the amplitude spectrum of the original sound energy. (5) The amplitude from each subband energy is recovered and integrated to recover the original sound to obtain a PR sound (le-PR) that preserves the two-stage spectra.

**Figure 3.**
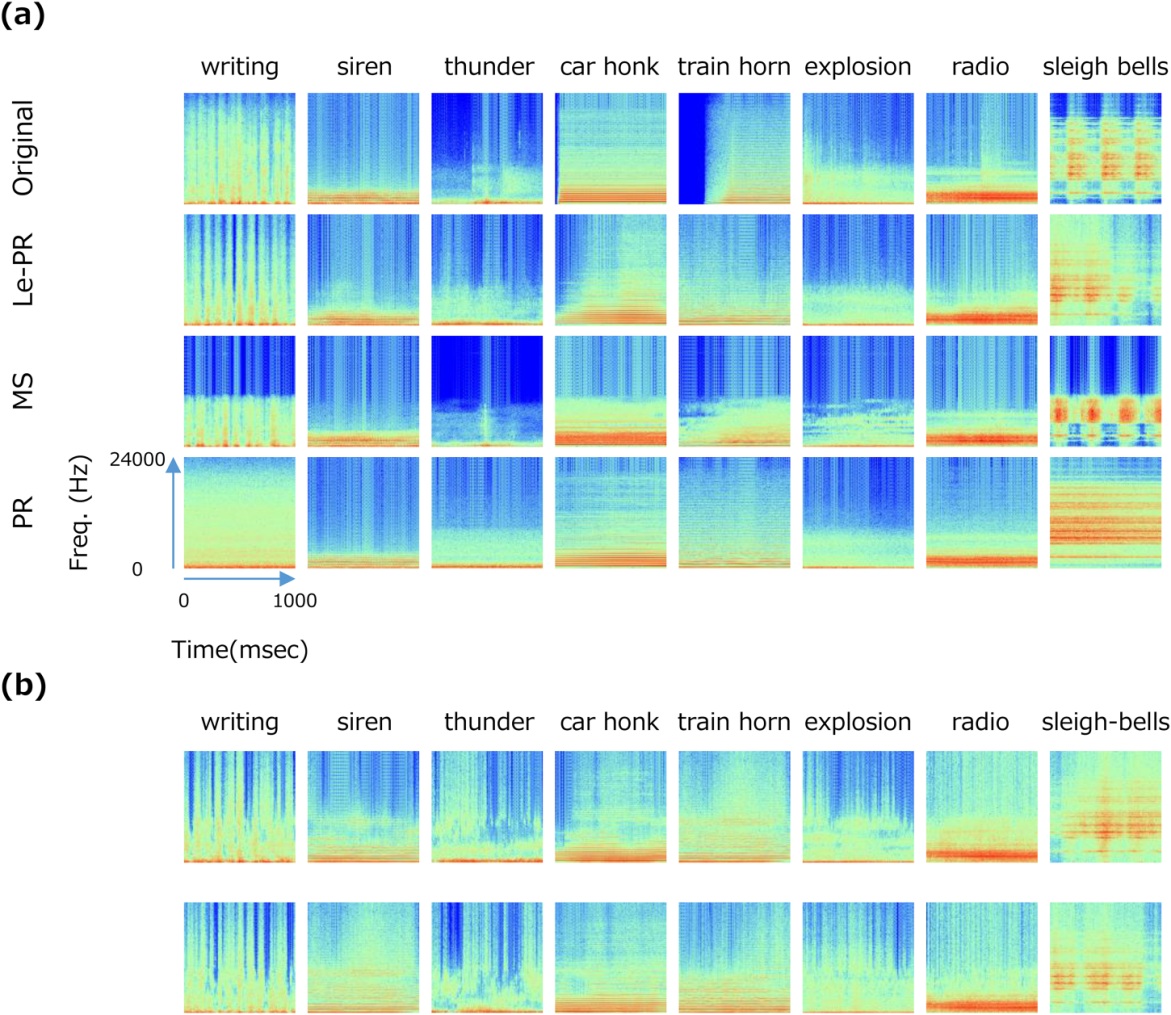
(a) Example spectrograms of the original sounds and three types of synthesized sounds. From top to bottom, the results are shown for the original sounds, le-PR, PR, and the MS-synthesized sound, respectively. Each column shows the name of the original natural sounds. (b) Examples of le-PR spectrograms with different random phase spectra, respectively.

*Note*. PR = phase-randomized sound; le-PR = a phase-randomized sound that preserves the two-stage spectra; MS = McDermott–Simoncelli.

Figure 3(a) shows examples of spectrograms of the original natural sounds (top) and three types of synthetic sounds. Our casual observations indicated that the MS synthesis and the two-stage spectrally preserved PR sound (le-PR) were perceptually similar to the original for many sounds. On the other hand, PR sounds with only a linear spectrum were often perceived as monotonous bandpass noise. Figure 3(b) shows spectrograms for several exemplars of le-PR sounds generated from different seeds of randomization.

### Psychophysical Experiment

In a simple psychophysical rating experiment, we tested how perceptually similar, or successfully synthesized, two-stage spectrally preserved noise sounds (le-PR) are to the original sounds. We also obtained ratings for two other types of sound synthesis, namely MS synthesis and spectrally matched noise (PR). Here, MS synthesis was performed using the Toolbox provided by McDermott's lab with the recommended parameters: subband variance, envelope mean, envelope variance, envelope skewness, envelope correlation, modulation power, and C1 correlation.

## Methods

### Apparatus

Auditory stimuli were presented to both ears at 70 dB SPL from a Sennheiser HD280 PRO via a PC-controlled Komplete Audio 1 (sampling rate 48 kHz, 24-bit D/A converter).

### Observers

Eight naïve participants without hearing impairment and two authors (mean age 23.3 years) participated in the experiment. All participants were nonprofessionals who were unskilled listeners and naïve about the purpose of the experiment. All experiments were conducted in accordance with the Declaration of Helsinki and with the permission of the Ethical Review Committee for Experimental Research on Human Subjects, Graduate School of Arts and Sciences, the University of Tokyo. All observers provided filled informed consent forms.

### Stimuli

**Figure 4.**
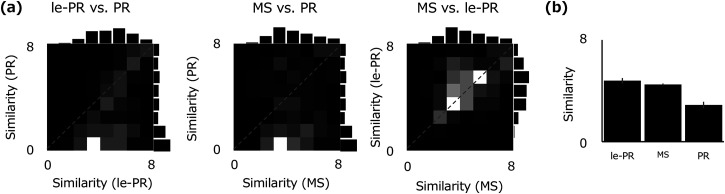
The perceptual similarity rating for the three types of synthesized sounds to the original natural sound: the MS statistics, the two-stage spectrum (le-PR), and the linear spectrum (PR). (a) Joint histograms of similarity ratings between different types of synthetic sounds. Each panel shows the comparison in ratings for le-PR versus PR (left), MS versus PR (middle), and MS versus le-PR (right). (b) Similarity ratings averaged across 120 natural sounds. Error bars represent ±1 SEM between participants.

*Note*. PR = phase-randomized sound; le-PR = a phase-randomized sound that preserves the two-stage spectra; MS = McDermott–Simoncelli; SEM = standard error of the mean.

The original auditory stimuli were 120 textural sounds related to a variety of natural events, including writing on paper, sleigh bells, thunderstorms, car horns, radio noise, sirens, and so on (see Supplemental Material). These were digital audio data downloaded from an online repository. Each sound was converted to mono, resampled at 48,000 Hz, and normalized to a fixed rms amplitude. For each of these natural sounds, three classes of synthetics sounds were generated: a synthesis based on MS statistics, PR sounds in a two-stage spectrum (le-PR, Figure 4), and PR sounds in the spectrogram. The MS-synthesized sounds were generated using 5 s segments from the original sound, as required by the algorithm, while the le-PR and PR sounds were generated using 3 s segments. In the experiment, 1 s segments were extracted so that one can easily recognize the sound category.

### Procedure

In each trial, the original natural sound and its synthesized sound were presented for 1 s with a 1 s silence in between. The type of synthesized sound was chosen at random. The participants rated how similar the synthesized sound was to the original natural sound by pressing a button on a 9-point scale: the same or very similar to the original (8), close to the original but slightly degraded (7), close to the original but a little synthesized (6), clearly heard as a synthetic sound (5), clearly synthetic and degraded sound quality (4), recognizable original but clearly unsuccessful synthesis (3), recognizable synthesis of the original but also noise (2), closer to a noise than to the synthesis of the original sound (1), a noise unrelated to the original (0). Note that all of these instructions were given in Japanese. Participants were also instructed not to use mere apparent loudness as a cue. To establish rating criteria, participants were presented with the full set of original sounds in random order immediately before the experiment. All participants rated each stimulus 3 times, and the average was used for analysis.

## Results

Figure 4a shows a joint histogram for the distribution of similarity ratings to the original sound for each of the three types of synthetic sound pairs (MS vs. le-PR, le-PR vs. PR, MS vs. PR). The brightness of each cell represents the relative frequency of responses. The gray diagonal line indicates that the ratings for the two types of synthesized sounds are equivalent; the area below this line indicates that the synthetic sound on the horizontal axis was more similar to the original sound than the synthetic sound on the vertical axis.

The two joint histograms on the left show that PR sounds with only a first-order linear spectrum preserved are often perceived as mere band-passed noise (i.e., rating of 0) and are rated as far less similar to the original compared to the other two synthesis types. While synthetic sounds with a two-stage spectral model (le-PR) are rarely less similar than PR sounds, some MS-synthesized sounds are rated as a little less similar than PR sounds. The joint histogram on the right shows that the ratings are similar between le-PR sounds and MS sounds. Figure 4b shows the mean ratings for 120 stimuli. The synthesized sounds by MS and le-PR are clearly more similar to the original sound than the PR sounds, and le-PR is slightly better than MS. One-way repeated-measure ANOVA showed a significant main effect of synthesis type, *F*(2, 27) = 8.6992, *p* < .00005. Two-sided *t*-tests showed significant differences for all combinations: le-PR versus MS, *t*(9) = 2.351, *p* < .05, le-PR versus PR, *t*(9) = 16.659, *p* < .0000001, and MS versus PR, *t*(9) = 7.877, *p* < .0001. This suggests that the perceptual quality of the synthesis based on the two-stage spectrum (le-PR) is comparable with, or slightly better than, the MS synthesis.

## Discussion

The present study proposed a simple model that describes the perception of sound textures in terms of first-stage (one-dimensional [1D]) and second-stage (2D) amplitude spectra. Psychophysical examination with 120 natural sounds revealed that synthetic-noise sounds that preserve the two-stage spectra of the original perceptually mimic the original sounds with a quality comparable to the sounds synthesized by MS’s statistics. These results support the idea that the perception of sound texture can be accounted for by two-stage spectral representations.

As mentioned earlier, the two-stage spectral model is analogous to a part of the model of MS statistics. The MS model employs sound statistics in terms of subband amplitudes, moments of energy, and correlations between energy or modulation filter outputs. Our two-stage model virtually represents these as amplitude spectra in the Fourier domain. However, the two-stage spectral model omits several classes of high-level statistics such as C1 and C2 correlations (McDermott & Simoncelli, 2011), and it has a simpler architecture than the MS model. Our model has many more parameters (i.e., amplitude data) than the MS model, and it is capable of representing the spectral structure of the sound with much higher accuracy than the MS model. Given this, it is not surprising that the synthetic sound based on our model (le-PR) was perceptually similar to the original sound with equal or slightly higher quality than the synthetic sounds based on MS statistics. It is also possible that MS-synthesized sounds that we generated with the recommended McDermott-lab toolbox were somehow degraded as compared to those generated with codes originally used in McDermott and Simoncelli (2011). In addition, the high performance of our model may be partially ascribed to the fact that we selected a wide range of textural sound samples without any particular criterion. In the development of the MS model, sound samples, which cannot be successfully synthesized without considering specific statistics such as C1 and C2 correlations, were intentionally introduced to illustrate the effects of various statistics (McDermott & Simoncelli, 2011). Our experimental results might have been different if such challenging samples had been included. Should our interpretation prove correct, our results would also suggest that spectral features assumed in the present model would be sufficient to model the perceptual representation of the majority of sounds in nature. As noted below, the MS model might attempt to consider higher-order features which are not relevant for sounds that are perceived as “textural sounds.”

Some sounds that were successfully synthesized with the two-stage spectral model were repetitive, such as writing on paper, thunder, sirens, and sleigh bells. On the other hand, some sounds, such as a knocking on the door, a baby's cry, and the opening and closing of a door, were not synthesized successfully by either the two-stage spectral model or the MS model. These poorly synthesized sounds may have contained some higher-order acoustic features. Such sounds may not be considered “sound textures,” but it is difficult to objectively define what stimuli are texture-like, either in vision or audition. In vision research, textural images are ambiguously defined as repetitions of similar patterns, but there are many images that are perceived as textural even if the lower-order image features are not spatially uniform. Similarly, sounds that are considered textural do not always consist of temporally stationary statistics. Kurosawa et al. (2021) recently examined “what kinds of images are perceived as textures” by using a wide range of natural images, including objects and scenes. Their results revealed a robust law that PS synthesis succeeds for natural images that are perceptually classified as “textures” by observers and fail for those that are not. This suggests that the image features assumed in the PS model are sufficient to describe the perception of “texture” and that images mischaracterized by the PS statistics are not processed by the visual system as “textures.” If we can define a domain for sound texture as well, we may be able to indicate what information is sufficient for describing auditory texture perception. Unlike the case of visual texture, however, it is unclear whether humans are able to easily distinguish between “textural” and “no-textural” sounds.

The two-stage spectral model of auditory texture perception has an analogous structure to the two-stage spectral model of visual texture (Okada & Motoyoshi, 2021), with the only difference being the dimensionality of the spectral space. Both models assume that texture perception is essentially based on two-stage filtering or convolution, suggesting a common computational principle in visual and auditory neural processing. The multistage convolution in the visual and auditory texture models is consistent with the basic scheme of image processing in the early visual cortex (Baker & Mareschal, 2001; Freeman & Simoncelli, 2011; Hubel & Wiesel, 1968; Ziemba et al., 2016; Zipser et al., 1996), and with sound processing in the auditory system as well (Baumann et al., 2011; Chi et al., 2005; Greenwood, 1990; Joris et al., 2004; Rodríguez et al., 2010). Specifically, it is well known that the auditory cortex has neurons that are selectively sensitive to particular time-frequency modulations (Depireux et al., 2001; Kowalski et al., 1996a, 1996b). The two-stage spectral analysis is also consistent with computations in deep neural networks based on convolution and pooling within multiple layers (Fukushima & Miyake, 1982; LeCun et al., 2015; Riesenhuber & Poggio, 2000), as well as with wavelet scattering networks used to compute translation-invariant image representations for classification (Bruna and Mallat, 2013; Mallat, 2012). We expect that such multiorder spectral analysis would be a general principle in low-level sensory information processing, including other modalities such as touch.

It should be noted that two-stage spectral analyses are still local in space (vision) and in time (audition), and the perception of a texture is determined by the summary (e.g., average) of these local signals over a particular spatial (vision) or temporal (audition) range. The amplitude spectra in our model and statistics in the MS model correspond to such summary representations. Thus, our model and the MS model implicitly assume an additional mechanism that temporally integrates (sums) the outputs of two-stage analyzers. For visual texture, such a summarization mechanism is known to exist in V4 (Freeman & Simoncelli, 2011; Okazawa et al., 2015; Ziemba et al., 2016), where neurons respond to particular image statistics within large spatial receptive fields. Based on these findings, it is expected that analogous neural mechanisms in the high-level auditory cortex would encode the statistical property of a sound over a long temporal period (cf. Feather & McDermott, 2018; Norman-Haignere & McDermott, 2018). However, it is unclear how such a mechanism ought to be implemented nor how long the temporal integration period should be.

## Supplemental Material

sj-docx-1-ipe-10.1177_20416695231157349 - Supplemental material for A two-stage spectral model for sound texture perception: Synthesis and psychophysics Click here for additional data file. Supplemental material, sj-docx-1-ipe-10.1177_20416695231157349 for A two-stage spectral model for sound texture perception: Synthesis and psychophysics by Hironori Maruyama, Kosuke Okada and Isamu Motoyoshi in i-Perception

sj-wav-2-ipe-10.1177_20416695231157349 - Supplemental material for A two-stage spectral model for sound texture perception: Synthesis and psychophysics Click here for additional data file. Supplemental material, sj-wav-2-ipe-10.1177_20416695231157349 for A two-stage spectral model for sound texture perception: Synthesis and psychophysics by Hironori Maruyama, Kosuke Okada and Isamu Motoyoshi in i-Perception

sj-wav-3-ipe-10.1177_20416695231157349 - Supplemental material for A two-stage spectral model for sound texture perception: Synthesis and psychophysics Click here for additional data file. Supplemental material, sj-wav-3-ipe-10.1177_20416695231157349 for A two-stage spectral model for sound texture perception: Synthesis and psychophysics by Hironori Maruyama, Kosuke Okada and Isamu Motoyoshi in i-Perception

sj-wav-4-ipe-10.1177_20416695231157349 - Supplemental material for A two-stage spectral model for sound texture perception: Synthesis and psychophysics Click here for additional data file. Supplemental material, sj-wav-4-ipe-10.1177_20416695231157349 for A two-stage spectral model for sound texture perception: Synthesis and psychophysics by Hironori Maruyama, Kosuke Okada and Isamu Motoyoshi in i-Perception

sj-wav-5-ipe-10.1177_20416695231157349 - Supplemental material for A two-stage spectral model for sound texture perception: Synthesis and psychophysics Click here for additional data file. Supplemental material, sj-wav-5-ipe-10.1177_20416695231157349 for A two-stage spectral model for sound texture perception: Synthesis and psychophysics by Hironori Maruyama, Kosuke Okada and Isamu Motoyoshi in i-Perception

sj-wav-6-ipe-10.1177_20416695231157349 - Supplemental material for A two-stage spectral model for sound texture perception: Synthesis and psychophysics Click here for additional data file. Supplemental material, sj-wav-6-ipe-10.1177_20416695231157349 for A two-stage spectral model for sound texture perception: Synthesis and psychophysics by Hironori Maruyama, Kosuke Okada and Isamu Motoyoshi in i-Perception

sj-wav-7-ipe-10.1177_20416695231157349 - Supplemental material for A two-stage spectral model for sound texture perception: Synthesis and psychophysics Click here for additional data file. Supplemental material, sj-wav-7-ipe-10.1177_20416695231157349 for A two-stage spectral model for sound texture perception: Synthesis and psychophysics by Hironori Maruyama, Kosuke Okada and Isamu Motoyoshi in i-Perception

sj-wav-8-ipe-10.1177_20416695231157349 - Supplemental material for A two-stage spectral model for sound texture perception: Synthesis and psychophysics Click here for additional data file. Supplemental material, sj-wav-8-ipe-10.1177_20416695231157349 for A two-stage spectral model for sound texture perception: Synthesis and psychophysics by Hironori Maruyama, Kosuke Okada and Isamu Motoyoshi in i-Perception

sj-wav-9-ipe-10.1177_20416695231157349 - Supplemental material for A two-stage spectral model for sound texture perception: Synthesis and psychophysics Click here for additional data file. Supplemental material, sj-wav-9-ipe-10.1177_20416695231157349 for A two-stage spectral model for sound texture perception: Synthesis and psychophysics by Hironori Maruyama, Kosuke Okada and Isamu Motoyoshi in i-Perception

sj-wav-10-ipe-10.1177_20416695231157349 - Supplemental material for A two-stage spectral model for sound texture perception: Synthesis and psychophysics Click here for additional data file. Supplemental material, sj-wav-10-ipe-10.1177_20416695231157349 for A two-stage spectral model for sound texture perception: Synthesis and psychophysics by Hironori Maruyama, Kosuke Okada and Isamu Motoyoshi in i-Perception

sj-wav-11-ipe-10.1177_20416695231157349 - Supplemental material for A two-stage spectral model for sound texture perception: Synthesis and psychophysics Click here for additional data file. Supplemental material, sj-wav-11-ipe-10.1177_20416695231157349 for A two-stage spectral model for sound texture perception: Synthesis and psychophysics by Hironori Maruyama, Kosuke Okada and Isamu Motoyoshi in i-Perception

sj-wav-12-ipe-10.1177_20416695231157349 - Supplemental material for A two-stage spectral model for sound texture perception: Synthesis and psychophysics Click here for additional data file. Supplemental material, sj-wav-12-ipe-10.1177_20416695231157349 for A two-stage spectral model for sound texture perception: Synthesis and psychophysics by Hironori Maruyama, Kosuke Okada and Isamu Motoyoshi in i-Perception

sj-wav-13-ipe-10.1177_20416695231157349 - Supplemental material for A two-stage spectral model for sound texture perception: Synthesis and psychophysics Click here for additional data file. Supplemental material, sj-wav-13-ipe-10.1177_20416695231157349 for A two-stage spectral model for sound texture perception: Synthesis and psychophysics by Hironori Maruyama, Kosuke Okada and Isamu Motoyoshi in i-Perception

sj-wav-14-ipe-10.1177_20416695231157349 - Supplemental material for A two-stage spectral model for sound texture perception: Synthesis and psychophysics Click here for additional data file. Supplemental material, sj-wav-14-ipe-10.1177_20416695231157349 for A two-stage spectral model for sound texture perception: Synthesis and psychophysics by Hironori Maruyama, Kosuke Okada and Isamu Motoyoshi in i-Perception

sj-wav-15-ipe-10.1177_20416695231157349 - Supplemental material for A two-stage spectral model for sound texture perception: Synthesis and psychophysics Click here for additional data file. Supplemental material, sj-wav-15-ipe-10.1177_20416695231157349 for A two-stage spectral model for sound texture perception: Synthesis and psychophysics by Hironori Maruyama, Kosuke Okada and Isamu Motoyoshi in i-Perception

sj-wav-16-ipe-10.1177_20416695231157349 - Supplemental material for A two-stage spectral model for sound texture perception: Synthesis and psychophysics Click here for additional data file. Supplemental material, sj-wav-16-ipe-10.1177_20416695231157349 for A two-stage spectral model for sound texture perception: Synthesis and psychophysics by Hironori Maruyama, Kosuke Okada and Isamu Motoyoshi in i-Perception

sj-wav-17-ipe-10.1177_20416695231157349 - Supplemental material for A two-stage spectral model for sound texture perception: Synthesis and psychophysics Click here for additional data file. Supplemental material, sj-wav-17-ipe-10.1177_20416695231157349 for A two-stage spectral model for sound texture perception: Synthesis and psychophysics by Hironori Maruyama, Kosuke Okada and Isamu Motoyoshi in i-Perception

sj-wav-18-ipe-10.1177_20416695231157349 - Supplemental material for A two-stage spectral model for sound texture perception: Synthesis and psychophysics Click here for additional data file. Supplemental material, sj-wav-18-ipe-10.1177_20416695231157349 for A two-stage spectral model for sound texture perception: Synthesis and psychophysics by Hironori Maruyama, Kosuke Okada and Isamu Motoyoshi in i-Perception

sj-wav-19-ipe-10.1177_20416695231157349 - Supplemental material for A two-stage spectral model for sound texture perception: Synthesis and psychophysics Click here for additional data file. Supplemental material, sj-wav-19-ipe-10.1177_20416695231157349 for A two-stage spectral model for sound texture perception: Synthesis and psychophysics by Hironori Maruyama, Kosuke Okada and Isamu Motoyoshi in i-Perception

sj-wav-20-ipe-10.1177_20416695231157349 - Supplemental material for A two-stage spectral model for sound texture perception: Synthesis and psychophysics Click here for additional data file. Supplemental material, sj-wav-20-ipe-10.1177_20416695231157349 for A two-stage spectral model for sound texture perception: Synthesis and psychophysics by Hironori Maruyama, Kosuke Okada and Isamu Motoyoshi in i-Perception

sj-wav-21-ipe-10.1177_20416695231157349 - Supplemental material for A two-stage spectral model for sound texture perception: Synthesis and psychophysics Click here for additional data file. Supplemental material, sj-wav-21-ipe-10.1177_20416695231157349 for A two-stage spectral model for sound texture perception: Synthesis and psychophysics by Hironori Maruyama, Kosuke Okada and Isamu Motoyoshi in i-Perception

sj-wav-22-ipe-10.1177_20416695231157349 - Supplemental material for A two-stage spectral model for sound texture perception: Synthesis and psychophysics Click here for additional data file. Supplemental material, sj-wav-22-ipe-10.1177_20416695231157349 for A two-stage spectral model for sound texture perception: Synthesis and psychophysics by Hironori Maruyama, Kosuke Okada and Isamu Motoyoshi in i-Perception

sj-wav-23-ipe-10.1177_20416695231157349 - Supplemental material for A two-stage spectral model for sound texture perception: Synthesis and psychophysics Click here for additional data file. Supplemental material, sj-wav-23-ipe-10.1177_20416695231157349 for A two-stage spectral model for sound texture perception: Synthesis and psychophysics by Hironori Maruyama, Kosuke Okada and Isamu Motoyoshi in i-Perception

sj-wav-24-ipe-10.1177_20416695231157349 - Supplemental material for A two-stage spectral model for sound texture perception: Synthesis and psychophysics Click here for additional data file. Supplemental material, sj-wav-24-ipe-10.1177_20416695231157349 for A two-stage spectral model for sound texture perception: Synthesis and psychophysics by Hironori Maruyama, Kosuke Okada and Isamu Motoyoshi in i-Perception

sj-wav-25-ipe-10.1177_20416695231157349 - Supplemental material for A two-stage spectral model for sound texture perception: Synthesis and psychophysics Click here for additional data file. Supplemental material, sj-wav-25-ipe-10.1177_20416695231157349 for A two-stage spectral model for sound texture perception: Synthesis and psychophysics by Hironori Maruyama, Kosuke Okada and Isamu Motoyoshi in i-Perception

sj-wav-26-ipe-10.1177_20416695231157349 - Supplemental material for A two-stage spectral model for sound texture perception: Synthesis and psychophysics Click here for additional data file. Supplemental material, sj-wav-26-ipe-10.1177_20416695231157349 for A two-stage spectral model for sound texture perception: Synthesis and psychophysics by Hironori Maruyama, Kosuke Okada and Isamu Motoyoshi in i-Perception

sj-wav-27-ipe-10.1177_20416695231157349 - Supplemental material for A two-stage spectral model for sound texture perception: Synthesis and psychophysics Click here for additional data file. Supplemental material, sj-wav-27-ipe-10.1177_20416695231157349 for A two-stage spectral model for sound texture perception: Synthesis and psychophysics by Hironori Maruyama, Kosuke Okada and Isamu Motoyoshi in i-Perception

sj-wav-28-ipe-10.1177_20416695231157349 - Supplemental material for A two-stage spectral model for sound texture perception: Synthesis and psychophysics Click here for additional data file. Supplemental material, sj-wav-28-ipe-10.1177_20416695231157349 for A two-stage spectral model for sound texture perception: Synthesis and psychophysics by Hironori Maruyama, Kosuke Okada and Isamu Motoyoshi in i-Perception

sj-wav-29-ipe-10.1177_20416695231157349 - Supplemental material for A two-stage spectral model for sound texture perception: Synthesis and psychophysics Click here for additional data file. Supplemental material, sj-wav-29-ipe-10.1177_20416695231157349 for A two-stage spectral model for sound texture perception: Synthesis and psychophysics by Hironori Maruyama, Kosuke Okada and Isamu Motoyoshi in i-Perception

sj-wav-30-ipe-10.1177_20416695231157349 - Supplemental material for A two-stage spectral model for sound texture perception: Synthesis and psychophysics Click here for additional data file. Supplemental material, sj-wav-30-ipe-10.1177_20416695231157349 for A two-stage spectral model for sound texture perception: Synthesis and psychophysics by Hironori Maruyama, Kosuke Okada and Isamu Motoyoshi in i-Perception

sj-wav-31-ipe-10.1177_20416695231157349 - Supplemental material for A two-stage spectral model for sound texture perception: Synthesis and psychophysics Click here for additional data file. Supplemental material, sj-wav-31-ipe-10.1177_20416695231157349 for A two-stage spectral model for sound texture perception: Synthesis and psychophysics by Hironori Maruyama, Kosuke Okada and Isamu Motoyoshi in i-Perception

sj-wav-32-ipe-10.1177_20416695231157349 - Supplemental material for A two-stage spectral model for sound texture perception: Synthesis and psychophysics Click here for additional data file. Supplemental material, sj-wav-32-ipe-10.1177_20416695231157349 for A two-stage spectral model for sound texture perception: Synthesis and psychophysics by Hironori Maruyama, Kosuke Okada and Isamu Motoyoshi in i-Perception

sj-wav-33-ipe-10.1177_20416695231157349 - Supplemental material for A two-stage spectral model for sound texture perception: Synthesis and psychophysics Click here for additional data file. Supplemental material, sj-wav-33-ipe-10.1177_20416695231157349 for A two-stage spectral model for sound texture perception: Synthesis and psychophysics by Hironori Maruyama, Kosuke Okada and Isamu Motoyoshi in i-Perception

sj-txt-34-ipe-10.1177_20416695231157349 - Supplemental material for A two-stage spectral model for sound texture perception: Synthesis and psychophysics Click here for additional data file. Supplemental material, sj-txt-34-ipe-10.1177_20416695231157349 for A two-stage spectral model for sound texture perception: Synthesis and psychophysics by Hironori Maruyama, Kosuke Okada and Isamu Motoyoshi in i-Perception

sj-m-35-ipe-10.1177_20416695231157349 - Supplemental material for A two-stage spectral model for sound texture perception: Synthesis and psychophysics Click here for additional data file. Supplemental material, sj-m-35-ipe-10.1177_20416695231157349 for A two-stage spectral model for sound texture perception: Synthesis and psychophysics by Hironori Maruyama, Kosuke Okada and Isamu Motoyoshi in i-Perception

sj-m-36-ipe-10.1177_20416695231157349 - Supplemental material for A two-stage spectral model for sound texture perception: Synthesis and psychophysics Click here for additional data file. Supplemental material, sj-m-36-ipe-10.1177_20416695231157349 for A two-stage spectral model for sound texture perception: Synthesis and psychophysics by Hironori Maruyama, Kosuke Okada and Isamu Motoyoshi in i-Perception
