# Supplementary material for: A two-stage spectral model for sound texture perception: Synthesis and psychophysics
Source: Iperception. 2023 Feb 22;14(1):20416695231157349. doi: 10.1177/20416695231157349 (PMC9950610; doi:10.1177/20416695231157349)
Supplement: sj-docx-1-ipe-10.1177_20416695231157349 - Supplemental material for A two-stage spectral model for sound texture perception: Synthesis and psychophysics [file sj-docx-1-ipe-10.1177_20416695231157349.docx]

Supplementary material

S1: Demo sounds Demo sounds used in our experiment

The demo samples are included: original sounds, synthesized sounds based on a two-stage spectral model (linear-Energy phase randomized sounds: le-PR), synthesized sounds preserving only the linear spectrum (phase randomized sounds: PR), and synthesized sounds using McDermott-Simoncelli's method (MS-synthesized sounds: MS). We have attached 10 files of each of the four types, for a total of 40 files. each files are named OG_1000_***.wav as the original sounds, ePR_1000_***.wav as the le-PR synthesized sounds, PR_1000_***.wav as the PR synthesized sounds and MS_1000_***.wav as the MS synthesized sounds .

Original sounds

OG_1000_car_honk.wav

OG_1000_explosion.wav

OG_1000_radio.wav

OG_1000_siren.wav

OG_1000_sleigh bells.wav

OG_1000_thunder.wav

OG_1000_train horn.wav

OG_1000_writing.wav

linear-Energy phase randomized sounds

le-PR_1000_car_honk.wav

le-PR_1000_explosion.wav

le-PR_1000_radio.wav

le-PR_1000_siren.wav

le-PR_1000_sleigh bells.wav

le-PR_1000_thunder.wav

le-PR_1000_train horn.wav

le-PR_1000_writing.wav

phase randomized sounds

PR_1000_car_honk.wav

PR_1000_explosion.wav

PR_1000_radio.wav

PR_1000_siren.wav

PR_1000_sleigh bells.wav

PR_1000_thunder.wav

PR_1000_train horn.wav

PR_1000_writing.wav

phase randomized sounds

MS_1000_car_honk.wav

MS_1000_explosion.wav

MS_1000_radio.wav

MS_1000_siren.wav

MS_1000_sleigh bells.wav

MS_1000_thunder.wav

MS_1000_train horn.wav

MS_1000_writing.wav

S2: Title list of sounds used in the experiment

walk

wind

Birdsong

Ventilation fan

Countdown

Fire

Underwater

Flipping through a book

Radio noise

Ambulance sirens

Cicada chirping

Crumpling paper

Loud bells

Fireworks

Fireworks

Vacuum cleaner suction

Railroad crossing

Forest

Writing on paper

Chewing

Thunderstorm

Human voice

Warnings

Rope rubbing

Thunderstorm

Fan

Explosion

Bells

Flowing water

Sandstorm

Flowing Water

Flowing Water

Explosion

Diving into water

Thunder

Thunder

Explosion

Writing on paper

Birdsong

Car horns

Clock hands

Association bells

Dog chirps

Crickets chirping

Swords rubbing

Sparks

Random Noise

Missile

Writing on paper

Crumpling paper

Turn over a piece of paper

Steam whistle

The sound of a car horn

Police car

Echoing clock hands

The chirping of birds

Fireworks going off

The sound of gunshots

The sound of cows

The bellowing of cows

Seat belts

Flowing water

Writing on paper

Tree falls down

Breathing after drinking

Walking on the floor

Plates hitting each other

Sipping water

Metal falling

Metal falling

Doors opening and closing

Knocking on the door

Howling

Beast roar

Cat meow

Typing noises

Opening a can of soda

Pepper mills

Pouring beer

Sipping water

The sound of a baby crying

Plastic falling off

Engine

Engine

Fried food

Deep breathing

Dishes clashing

Doorbell

Chewing sound

steam whistle

TV on/off

Fire

Door open/close

Woman yelling

The croaking of frogs

Dora

The sound of a beep

Ship's whistle

Laughter

Clapping and cheering

Dog barking

Radio noise

Kissing

The roar of a beast

Chips

Breathless

Air conditioner outdoor unit

Viscous mixture

Horse running

Radio noise

Car horn

Car door opening and closing

Wind

Pepper mills

Clapping and cheering

Laughter

Opening and closing of doors

Bell

Cars bumping into each other

Closing a large door
